# The Interactive Effects of Deficit Irrigation and *Bacillus pumilus* Inoculation on Growth and Physiology of Tomato Plant

**DOI:** 10.3390/plants12030670

**Published:** 2023-02-03

**Authors:** Jie Liu, Jiarui Zhang, Qimiao Shi, Xiangliang Liu, Zhen Yang, Pan Han, Jingjing Li, Zhenhua Wei, Tiantian Hu, Fulai Liu

**Affiliations:** 1 Key Laboratory of Agricultural Soil and Water Engineering in Arid and Semiarid Areas, Ministry of Education, Northwest A&F University, Xianyang 712100, China; 2 Department of Plant and Environmental Sciences, Faculty of Science, University of Copenhagen, Højbakkegaard Allé 13, 2630 Taastrup, Denmark

**Keywords:** water use efficiency, PGPR, leaf gas exchange, nutrition uptake

## Abstract

The effects of inoculating plant growth promoting rhizobacteria (PGPR) and soil water deficits on crop growth and physiology remain largely unknown. Here, the responses of leaf gas exchange, growth, and water use efficiency (WUE) of tomato plants to *Bacillus pumilus* (*B.p.*) inoculation under four irrigation strategies (I1-I4) were investigated in a greenhouse. Results showed that soil water deficits, especially at I4 (20%, *v*/*v*), significantly decreased leaf stomatal conductance (g_s_), transpiration rate (T_r_), and photosynthetic rate (A_n_), and the decrease of g_s_ and T_r_ were more pronounced than A_n_. Reduced irrigation regimes significantly lowered dry matter and plant water use both in the non-*B.p.* control and the *B.p.* plants, while reduced irrigation significantly increased plant WUE, and *B.p.* inoculation had little effect on this parameter. Synergistic effects of PGPR and deficit irrigation on leaf gas exchange, leaf abscisic acid content, and stomatal density were found in this study, and specifically, *B.p.* treated plants at I4 possessed the highest WUE at stomatal and leaf scales, suggesting that *B.p.* inoculation could optimize water use and partly alleviate the negative effects of soil water deficit. These findings provide useful information for effective irrigation management and the application of PGPR in agriculture in the future.

## 1. Introduction

Drought is one of the most important factors that threaten global crop production [1]. The impact of drought depends on crop species, as well as the intensity and duration of the stress [2]. Therefore, it is essential to understand how soil water deficits at different severities affect crop growth and physiology. Deficit irrigation (DI) is a strategy of saving water that involves irrigating the root zone with less water than required for crop evapotranspiration [3]; it optimizes crop water use efficiency (WUE) with an accepted reduction in yield and has been extensively studied throughout the world [4,5]. Under DI, plants can trigger root-to-shoot chemical signals, mainly abscisic acid (ABA), inducing partial stomatal closure and depressing leaf expansion growth, hereby decreasing stomatal conductance (g_s_) and transpiration rate (T_r_) [6]. DI reduces leaf gas exchange rates, while g_s_ and T_r_ are more sensitive to soil water deficits compared to photosynthetic rate (A_n_). Thus the plants often possess higher WUE under DI [7,8]. Moreover, hydraulic signaling and other phytohormones could also be involved in regulating stomatal behavior and photosynthetic activity under soil water deficits [2,9].

The microorganisms, mainly bacteria, that positively influence the growth and health of plants are called plant growth-promoting rhizobacteria (PGPR) [10]. PGPR application in agriculture has increased steadily in recent years, and they are expected to partly replace chemical fertilizers, pesticides, and other growth regulators in the future [11], providing an environmentally friendly solution for sustainable agricultural practices [12]. PGPR can directly or indirectly facilitate plant growth, either in vitro, in a greenhouse or under field conditions [13]. PGPR contains different kinds of bacteria communities, among which the gram-positive spore-forming *Bacillus* is one of the most promising PGPR, gaining increasing attention due to its inherent stability and extended shelf life [14]. *Bacillus* genus can promote plant growth by stimulating the synthesis of plant hormones such as 1-aminocyclopropane-1-carboxylate (ACC) deaminase, indoleacetic acid, and auxin and thus alleviating drought stress [15,16,17]. A variety of PGPR species, such as *Bacillus pumilus* and *Bacillus pallidus*, were able to induce abscisic acid (ABA) synthesis and effectively regulate stomatal movement and reduce water loss [18], sustaining plant growth in drying soil [15]. Therefore, it is of great interest to understand the interactive effect of deficit irrigation and PGPR inoculation on plant growth and physiology.

The ability of PGPR to improve plant nutrient utilization efficiency has been widely studied [19,20,21,22]. N_2_-fixing and P-solubilizing bacteria are important for plant nutrition in terms of increasing N and P uptake. Bacteria such as *Bacillus* are N-fixing and P-solubilizing microorganisms; they could enhance nutrient uptake and improve the growth and yield of crops [20,23]. Numerous studies have reported that PGPR not only promotes crop growth but also enhances the uptake efficiency of N and P, reducing the potential loss of these nutrients to the environment [19,22,24,25].

Tomato plants have a great need for water and are sensitive to water deficit [8,26]. For example, soil water deficit during the vegetative growth stage depresses leaf gas exchange rates and plant growth [8]. Soil water deficit during the fruit development stage decreases final yield [26]. In this study, tomato plants were grown with or without PGPR inoculation and were subjected to four irrigation regimes (I1-irrigated to 35% (*v*/*v*) soil water content (SWC); I2-irrigated to 30% SWC; I3-irrigated to 25% SWC; and I4-irrigated to 20% SWC). The objective was to investigate whether PGPR could alleviate the negative effects of soil water deficits on the plant growth of tomato plants and to explore how soil water deficits at different severities would affect the efficiency of PGPR in promoting the performance of the crop.

## 2. Results

### 2.1. Leaf Gas Exchange

**Figure 1:**
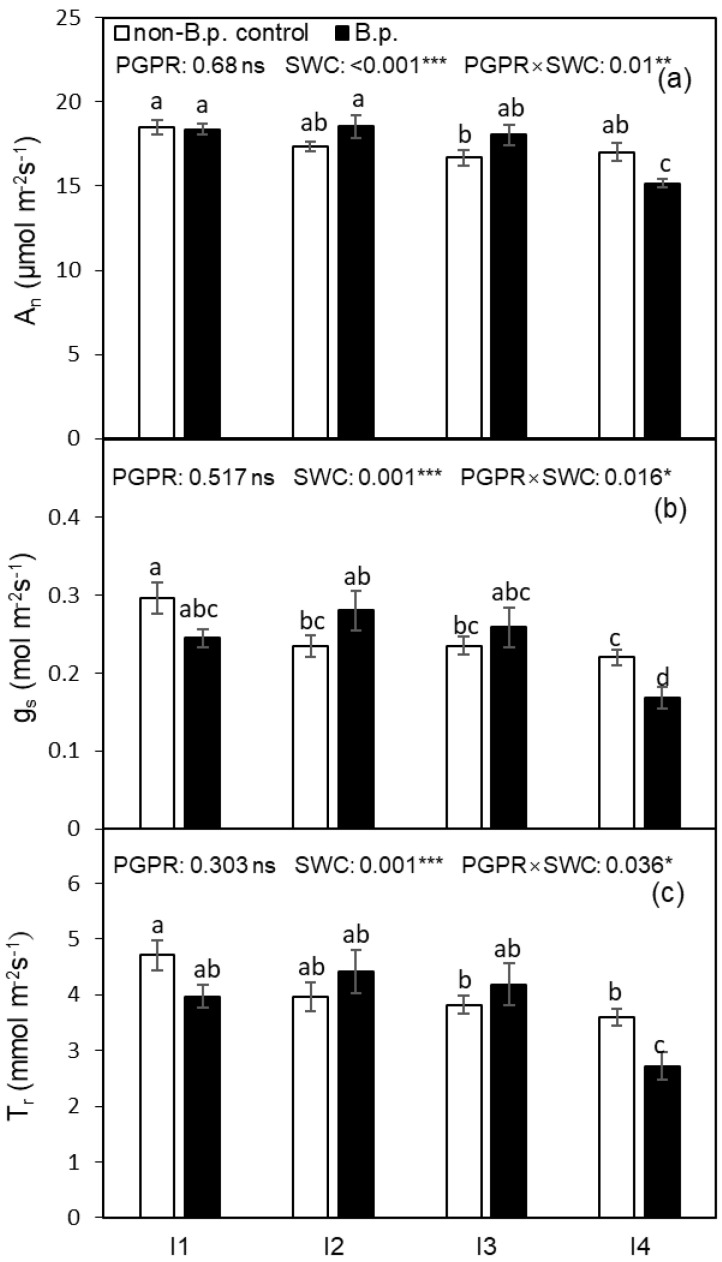
Photosynthesis rate (A_n_), stomatal conductance (g_s_), and transpiration rate (T_r_) of tomato leaf as affected by PGPR inoculation under four irrigation regimes (I1—irrigated to 35% (*v*/*v*) SWC; I2—irrigated to 30% SWC; I3—irrigated to 25% SWC; and I4—irrigated to 20% SWC). Different letters indicate a significant difference among treatments based on Tukey’s honestly significant different test after Two-way ANOVA. Error bars indicate the standard error of the means (SE), n = 4. (**a**–**c**) in the figure are corresponding to A_n_, g_s_ and T_r_, respectively. ns indicates the effect was not statistically significant at *p* < 0.05 level; *, ** and *** indicate significance levels at *p* < 0.05, *p* < 0.01 and *p* < 0.001, respectively.

The leaf gas exchange rates, including A_n_, g_s_, and T_r_, were not affected by *Bacillus pumilus* (*B.p.*) inoculation before the onset of the irrigation treatment (Table 1) but were significantly affected by the soil water content (SWC) and the PGPR × SWC interaction (Figure 1) after starting the irrigation regimes. Compared to plants free from *B.p.* inoculation (non-*B.p.* control), *B.p.* treated plants showed greater reductions in A_n_, g_s_ and, T_r_ under I4 (20%, *v*/*v*). Compared to I1 (35%, *v*/*v*) treatment, the A_n_, g_s_ and, T_r_ of I4 plants decreased by 12.8%, 28.3%, and 27.4%, respectively, showing the g_s_ and T_r_ were more sensitive to the water deficit than A_n_.

**Figure 2:**
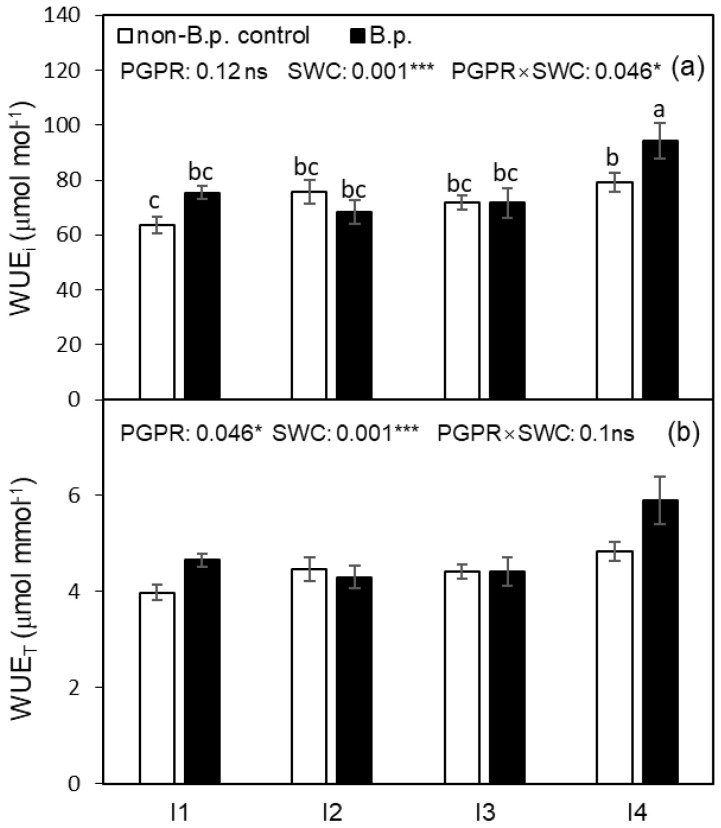
Intrinsic water use efficiency (WUE_i_) and instantaneous water use efficiency (WUE_T_) of tomato leaf as affected by PGPR inoculation under four irrigation regimes. Different letters indicate a significant difference among treatments based on a Tukey’s honestly significant different test after Two-way ANOVA. Error bars indicate the standard error of the means (SE), n = 4. (**a**,**b**) in the figure are corresponding to WUE_i_, and WUE_T_, respectively. ns indicates the effect was not statistically significant at *p* < 0.05 level; * and *** indicate significance levels at *p* < 0.05 and *p* < 0.001, respectively.

The intrinsic water use efficiency (WUE_i_) was significantly affected by SWC and PGPR × SWC interaction (Figure 2a). The effect of PGPR on WUE_i_ was not significant under I1 (35%, *v*/*v*), I2 (30%, *v*/*v*), and I3 (25%, *v*/*v*), while under I4 (20%, *v*/*v*), WUE_i_ was significantly improved by *B.p.* inoculation. The instantaneous water use efficiency (WUE_T_) was significantly affected by PGPR and SWC (Figure 2b). *B.p.* inoculation significantly improved WUE_T_. Regardless of PGPR inoculation, plants grown under I4 possessed higher WUE_i_ and WUE_T_ compared to those grown under I1.

### 2.2. Leaf Abscisic Acid Concentration, Stomatal Density and Leaf Water Potential

Leaf abscisic acid concentration ([ABA]_leaf_) was significantly affected by PGPR, SWC, as well as their interaction (Table 2). As predicted, [ABA]_leaf_ in I4 plants was higher than that in I1 plants, increased by 47.5% in non-*B.p.* control plants and 9.2% in *B.p.* ones, respectively. Interestingly, *B.p.* treated plants had higher [ABA] in the leaf than non-*B.p.* plants when SWC was at I1 and I2 (30%, *v*/*v*).

Stomatal density (SD) was not affected by PGPR (Table 1 and Table 2), but was significantly affected by SWC and PGPR × SWC interaction (Table 2). For non-*B.p.* control plants, there was no significant difference in SD among the four irrigation regimes, while for *B.p.* plants, SD was greater at more severe soil water deficit and was the greatest at I3 (25%, *v*/*v*).

Leaf water potential (Ψ_l_) was solely affected by SWC (Table 2). Plants grown under reduced irrigation regimes (I2, I3, and I4) had significantly lower Ψ_l_ compared to that under well-watered conditions (I1).

### 2.3. Leaf Area, Dry Matter and Specific Leaf Area

In relation to I1 and I2, I3 and I4 treatments significantly decreased leaf area (LA), leaf dry matter (DM_leaf_), and total dry matter (DM) (Table 2). The PGPR and PGPR × SWC had no evident effect on DM_leaf_, DM, LA, or SLA (Table 1 and Table 2).

### 2.4. Dry Matter Increment, Water Use and Plant Water Use Efficiency

Dry matter increment (ΔDM) and plant water use (WU) were only affected by SWC (Table 2). The maximum of ΔDM was obtained at I2 in both non-*B.p.* control and *B.p.* plants, with the most WU. Plant water use efficiency (WUE) was significantly improved by reduced SWC, and the maximum WUE was observed at I4, where plants possessed the minimal WU with a minor decrease of ΔDM (Table 2).

### 2.5. The N, P and K Contents in Leaf and Stem

SWC significantly affected the contents per kg dry weight of N, P, and K in both leaves and stems (Table 3). In relation to I1, I4 significantly depressed these parameters, while the differences in [N]_leaf_, [P]_leaf_ [K]_leaf_, and [K]_stem_ among I1, I2 and I3 were not significant, respectively. *B.p.* inoculation only significantly improved [N]_stem_ (Table 3). [P]_stem_ was also affected by PGPR × SWC interaction; compared to non-*B.p.* control, *B.p.* decreased [P]_stem_ under I3, while there was no significant difference in [P]_stem_ between non-*B.p.* control and *B.p.* plants under I1, I2 or I4 regimes, respectively (Table 3).

### 2.6. Nutrients Uptake and Nitrogen Use Efficiency

The total N, P, and K uptake of tomato plants were only significantly affected by SWC (Table 3). Compared to I1, the uptake of N, P, and K in I4 decreased by 25.9%, 36.5%, and 21.5%, respectively.

Nitrogen use efficiency (NUE) was only affected by SWC (Table 3), where reduced irrigation regime improved NUE. Compared to I1, I4 increased NUE by 12.1% and 22.5% in non-*B.p.* control and *B.p.* plants, respectively.

## 3. Discussion

### 3.1. Responses of Leaf Gas Exchange to PGPR Inoculation under Deficit Irrigation

In this study, plants grown under deficit irrigation, especially under I4 (20% SWC) regime, had lower A_n_, T_r_, and g_s_ than those under full irrigation (I1; Figure 1). As the transpiration process is more sensitive to water deficit than the photosynthetic process, thus crop can maintain a certain level of A_n_ in the water deficit state, resulting in an improved water use efficiency at leaf scales (Figure 2) [8,27,28]. It has been suggested that under low soil water content, the hydraulic water transport may cause localized lowering of leaf water potential in cells near the stomatal pores [29]. Alternatively, root system-produced chemical signals, such as abscisic acid (ABA), may be transported to the leaves via the xylem, inducing stomatal closure and hence lowering g_s_, T_r_, and plant water consumption [6]. Soil water deficits restrict roots from absorbing sufficient water to meet plant needs, resulting in a decline in leaf water potential, finally depressing leaf g_s_ and A_n_. Additionally, previous studies have shown that soil water deficits could affect stomatal morphology, thereby influencing the gas exchange process of plants [30]. Numerous studies have found that reduced irrigation significantly increases SD compared with full irrigation [31,32]. In the present study, SD decreased slightly with increasing the severity of soil water deficit under non-*B.p.* control, while under *B.p.*, reduced irrigation increased SD compared to FI, and this increase was most significant under the I3 regime (Table 2). Yan et al. and Liu et al. found a significant positive linear relationship between SD and leaf ABA, but this relationship was not seen in this study [32,33]. This may be due to the significant effect of PGPR alone on leaf ABA, as well as different nutrients, e.g., N and P status, of the tomato plants [31,33].

A recent study on potato plants showed that PGPR increased leaf A_n_, g_s_, and T_r_ at the early seedling stage but depressed these parameters gradually at the final harvest [34]. Higher WUE_i_ (A_n_/g_s_) can be achieved either through lower stomatal conductance or higher photosynthetic capacity, or both [35]. In the present study, the lowered A_n_, g_s_, and T_r_ and improved WUE_i_ and WUE_T_ by *B.p.* inoculation under I4 were observed (Figure 1; Figure 2). This could be due to plants with PGPR inoculation with *B.p.* more efficiently inducing stomatal closure and increasing the wax content of the leaf epidermis, thus reducing water loss and enhancing the drought tolerance of plants [36]. Our study revealed that compared to non-*B.p.* control, *B.p.* inoculation significantly promoted ABA synthesis when SWC at I1 and I2 (Table 2), yet the difference was not sufficient to cause a significant change of g_s_ (Figure 1b).

### 3.2. Response of Plant Growth and WUE to PGPR Inoculation under Deficit Irrigation

In this study, deficit irrigation regimes I3 and I4 significantly depressed tomato growth, including the reduction in DM and LA in relation to I1 (Table 2). Similar results were also reported in potato plants by Liu et al. [34]. The leaf expansion rate is very sensitive to soil water deficits [37], and the first morphological response of the plant to water deficits was lessening in LA [38]. Besides, here the WU and WUE of tomato plants were significantly affected by DI (Table 2). Previous studies suggested that DI increases the WUE of tomato plants [26,39,40]. Consistent with this, plant WUE was significantly greater at I3 or I4 than at I1; this was mainly due to the less pronounced decrease of DM (I3, 11.14%; I4, 13.09%) than WU (I3, 17.10%; I4, 28.19%) in relation to the I1 treatment (Table 2). We noticed that LA and DM between I1 and I2 plants were comparable (Table 2); this was mainly due to the fact that I2 was a mild DI strategy, which has a less negative effect on plant growth.

Literature suggests that PGPR inoculation could improve plant growth and dry matter accumulation [19,20]. In the present study, however, such stimulating effect of PGPR was not evident (Table 2). Recent studies have also demonstrated that *Bacillus* inoculation could improve plants WUE by up to 46% during water deficit (65% *θ_f_*) and rehydration (90% *θ_f_*) stages and could serve as an effective strategy to maintain crop yield under water-limited conditions [41]. This was not the case in the present study, where *B.p.* inoculation did not affect WUE. Explicitly, in the present study, the leaf gas exchange, plant growth, and WUE were mainly affected by the DI treatment rather than the PGPR *Bacillus pumilus*, consistent with our recent results [34]. The effectiveness of bio-inoculants on the enhancement of plant growth under stressful conditions could also be compromised by the low survival rate in the soil as well as competition with the indigenous community [42]. On the other hand, inoculation number and selected species or strong competition with native soil microorganisms could lead to non-competitive colonization in roots, especially under the DI strategy [34].

### 3.3. Response of the N, P and K Contents in Plant Organs and Nitrogen Use Efficiency to PGPR Inoculation under Deficit Irrigation

It is well documented that irrigation regimes and PGPR treatments significantly affected N accumulation in plant tissues [19]. Soil water deficits often reduce plant N uptake leading to a low N concentration in leaves and stems [43,44]. Consistent with this, the total N uptake of the I4 plants was less than that of I1, with reduced [N]_leaf_ and [N]_stem_ (Table 3). This might be related to the lowered value of the diffusion coefficient of both nitrate and ammonium under soil water deficit. Additionally, as the WU of plants under I4 was significantly lower than that under I1 (Table 2), this would limit the mass flow for N uptake, leading to the lowered [N]_leaf_ and [N]_stem_ in the I4 plants (Table 3).

Plant N use efficiency (NUE), as an indicator of N utilization for C acquisition in plants, is widely used in guiding agricultural production [45]. Studies have suggested that soil water deficits tend to lower plant NUE [8,46]. In the present study, NUE showed an increasing trend from I1 to I4 regardless of PGPR treatment (Table 3). This could be attributed to the more pronounced decrease of N accumulation compared to that for plant dry matter accumulation under soil water deficits (Table 2 and Table 3).

Evidence has indicated that soil water deficits could reduce the uptake of P and K in plants [47]. Likewise, here the deficit irrigation regimes also adversely affected P and K uptake of tomato plants (Table 3). It is notable that the magnitude of the influence differed between N and P; for instance, in relation to I1, I4 decreased [N]_leaf_ and [N]_stem_ by 15.37% and 8.42%, respectively, while decreased [P]_leaf_ and [P]_stem_ by 26.78% and 26.15%, respectively (Table 3).

Increased NUE in tomato, maize, and cucumber plants inoculated with PGPR have respectively been reported by Adesemoye et al. [25], Zeffa et al. [48], and Zhang et al. [49], while such positive effects on NUE by PGPR was not seen in our study, as *B.p.* inoculation had little effect on DM (Table 2) or total N uptake (Table 3). Masooda et al. (2020) reported that *B.p.* promoted the growth and N uptake of tomato plants [50]. In partial agreement with this, in our study, *B.p.* inoculation improved [N]_stem_ (Table 2), indicating *B.p.* has the potential to improve plant N status.

## 4. Materials and Methods

### 4.1. Site Description and Materials

**Figure 3:**
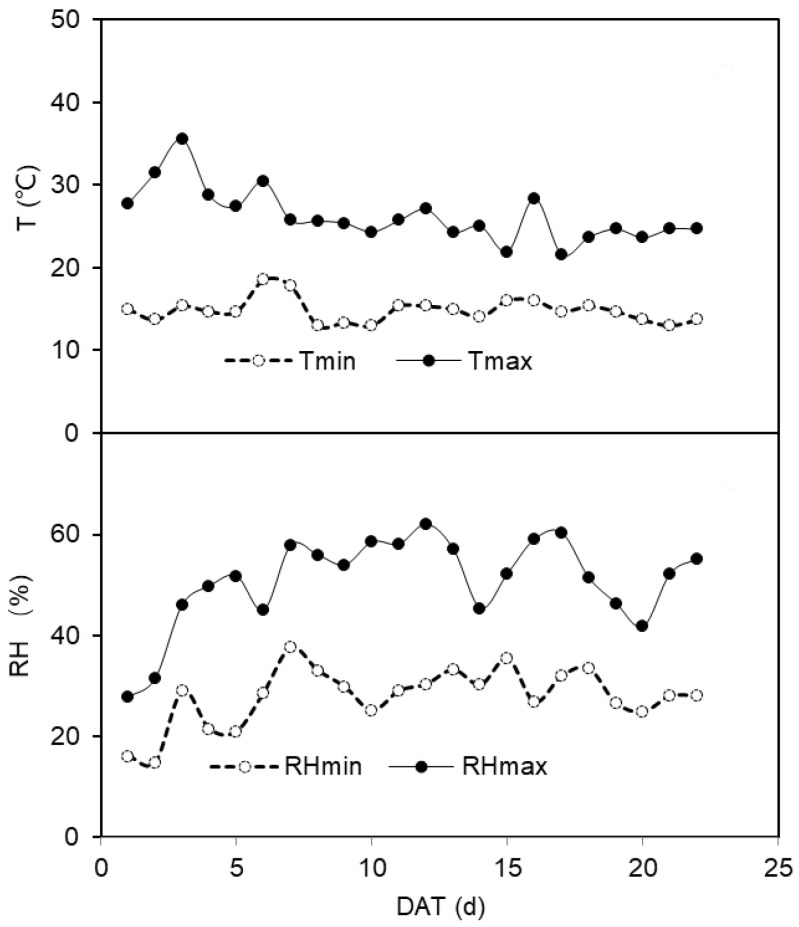
The maximum and minimum daily temperature (T, °C) and the daily maximum and minimum relative humidity (RH, %) in the greenhouse during the treatment period. Tmin and Tmax indicate the minimum and maximum daily temperature, respectively; RH min and RHmax indicate the minimum and maximum daily relative humidity, respectively.

The experiment was conducted in a glasshouse at Northwest A&F University, Yangling, Shaanxi, China (34°20″ N, 108°04″ E, and altitude of 521 m). Tomato seeds (Lycopersicon. esculentum Mill. Jinpeng 10, local cultivar) were sown in tray on 3rd December 2020 in the glasshouse. The climate conditions, including air temperature (T) and relative humidity (RH) in the glasshouse, were recorded by a Humidity & Temperature meter (TH-Logger) as shown in Figure 3. The photosynthetic active radiation (PAR) was supplied by sunlight plus high-pressure sodium lamps to keep it >500 μmol m^−2^ s^−1^. The soil used in the experiment was taken from the 0–20 cm layer in a local field and was classified as silty clay loam soil. The volumetric soil water content (SWC) at field capacity (*v*/*v*, *θ_f_*) was 31.2%, and the content of organic matter, rapid available N, and rapidly available K was 6.77 g kg^−1^, 127.72 mg kg^−1^, and 205 mg kg^−1^, respectively. To guarantee the a sufficient nutrition supply, 6 kg air-dried soil was mixed with 0.5 g N in the form of urea, 0.3 g K and, 0.24 g P in the form of KH_2_PO_4_ thoroughly before filling the pots (15 cm diameter and 30 cm depth).

### 4.2. Treatments

Thirty days after sowing, tomato seedlings at the three-leaf stage were uprooted from the plastic plug trays and carefully washed with deionized water; thereafter, roots were soaked for 4–5 h in a solution without (non-*B.p.*) or with *Bacillus pumilus* (*B.p.*) (10^8^ CFU mL^−1^). The *Bacillus pumilus* strain was provided by the Agricultural Culture Collection of China (ACCC 19290, Beijing, China). Fresh bacterial culture was prepared with the method described by Masood et al. [49]. Then the pre-soaked plants were separately transplanted into 6 L pots; each pot contained one plant. For plants pre-soaked in the solution with *Bacillus pumilus*, 1 mL of sterile water containing *Bacillus pumilus* (10^8^ CFU mL^−1^) was added to the planting holes; for plants pre-soaked in the solution without *Bacillus pumilus*, 1 mL of sterile water was added to the planting holes. Thereafter, the plants were irrigated to field capacity.

Two weeks later, 4 non-*B.p.* plants and 4 *B.p.* inoculated plants were harvested to investigate the effect of *B.p.* inoculation on plant growth at the early seedling stage. Since then, the average soil water content (%, *v*/*v*) in the pot was monitored at 4:00 pm every day by a time-domain reflectometer (TRASE; Soil Moisture Equipment Corporation, Santa Barbara, CA, USA) with probes (25 cm in length) installed vertically in the middle of the pot. The plants were subjected to four levels of irrigation regimes (I1—irrigated to 35% (*v*/*v*) SWC; I2—irrigated to 30% SWC; I3—irrigated to 25% SWC; and I4—irrigated to 20% SWC). At each irrigation event, the irrigation volume in a liter (L) was calculated as:

I = 5 × (θ_i_ − θ_mean_)

where 5 is the soil volume in the pots (L), θ_i_ is the SWC to which plants were irrigated (i.e., I1, I2, I3, and I4), and θ_mean_ is the mean SWC measured before irrigation [44].

**Figure 4:**
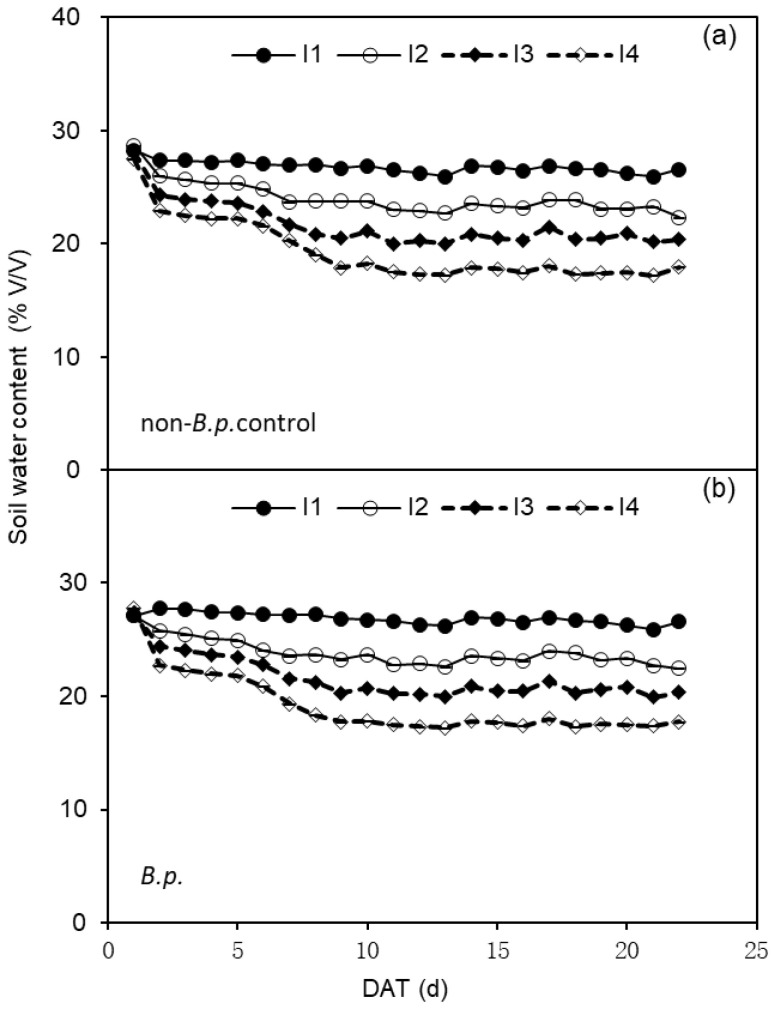
Change of soil water content (%, *v*/*v*) in the pots of tomato subjected to four irrigation regimes (I1—irrigated to 35% (*v*/*v*) SWC; I2—irrigated to 30% SWC; I3—irrigated to 25% SWC; and I4—irrigated to 20% SWC) without or with *Bacillus pumilus* (non-*B.p.* control or *B.p.*) inoculation. (**a**,**b**) in the figure are corresponding to SWC of non-*B.p.* control and *B.p.* plants, respectively.

The experiment setup was a complete randomized design with four replicates for each treatment resulting in 40 pots in total (8 plants at first harvest included). The irrigation treatment lasted for ca. 3 weeks; then all plants were harvested. During the irrigation treatment, the average soil water content in pots were displayed in Figure 4.

### 4.3. Measurements

#### 4.3.1. Leaf Gas Exchange

From the start of irrigation treatment, leaf gas exchange rates, including net photosynthetic rate (A_n_, μmol m^−2^ s^−1^), stomatal conductance (g_s_, mol m^−2^ s^−1^), and transpiration rate (T_r_, mmol m^−2^ s^−1^) were determined weekly. The measurement was performed on upper canopy fully expanded leaves between 9:00 and 11:00 am with a portable photosynthetic system (LiCor-6800, LI-Cor, NE, USA), at 25 °C chamber temperature, 1200 μmol m^−2^ s^−1^ photon flux density and 400 ppm CO_2_ concentration. Intrinsic and instantaneous water use efficiency, i.e., WUE_i_ and WUE_T_, were calculated as A_n_/g_s_ and A_n_/T_r_, respectively.

#### 4.3.2. Leaf Water Potential and ABA Concentration

Leaf water potential (Ψ_l_) was determined at the first harvest (two weeks after transplanting) and the final harvest (five weeks after transplanting) using a pressure chamber (Soil Moisture Equipment, SEC, Santa Barbara, CA, USA) on the same leaf where the gas exchange rates were measured. Immediately after the measurement, the leaf was wrapped in aluminum foils, then stored in a −80 °C refrigerator for subsequent determination of leaf abscisic acid content ([ABA]_leaf_). [ABA]_leaf_ was determined by enzyme-linked immunoassay (ELISA) following the protocol by [51].

#### 4.3.3. Dry Biomass, Leaf Area, Specific Leaf Area, Water Use and Water Use Efficiency

Plant dry biomass was estimated at the first harvest and the final harvest after drying at 70 °C in an oven to a constant weight. The difference between the DM at the final and the first harvest was the dry matter increment (ΔDM). At the final harvest, the total leaf area (LA, cm^2^) was measured with a portable leaf area meter (LI-3100, Inc. Lincoln, NE, USA), and the specific leaf area (SLA, cm^2^ g^−1^) was calculated as the ratio of LA to DM_leaf_. The plant water use (WU, L) during the irrigation period was estimated as the total volume of irrigation water plus the changes of soil water in the pots between the first and the final harvest. Plant water use efficiency (WUE) was calculated as the ratio of ΔDM to WU.

#### 4.3.4. Leaf Stomatal Density

Mature leaves for gas exchange measurement were selected to measure leaf stomatal density (SD, mm^−2^). The imprints of the upper and lower epidermis of the leaf were obtained using a silicone rubber gun. Specifically, the silicone rubber was evenly coated on the adaxial and abaxial leaf, then gently collected after it solidified. The colorless transparent nail polish was smeared evenly upon the silicone rubber surface, and after air drying, the epidermal imprints were attached to the microscopic slide using transparent tape (i.e., nail polish printing method). Then slides were photographed using a digital electron microscope (BA210Digital, Motic, Xiamen, China) with a connected image editing software (Leica Microsystems, version 2.5.0, CMS GmbH, Heerbrugg, Switzerland). Three images (calibrated size of 320 × 240 μm) were taken for each epidermal impression. The number of stomata for each image was counted through the ImageJ software (Version 1.51k, Wayne Rasband, National Institutes of Health, Bethesda, MD, USA, Java 1.6.0–24 (64 bit)), and SD was calculated as the number of stomata per mm^2^.

#### 4.3.5. Nutrient Contents and N Use Efficiency

The leaf and stem dry samples were grounded into a fine powder for analysis of total N, total P, and total K contents using a CHNS/O Elemental Analyser (Flash 2000, Thermo Fisher Scientific, Cambridge, UK). The plant nutrient uptake was estimated as the sum of each organ N, P, and K accumulation which was calculated as the multiplication of N, P and, K concentration with dry matter in leaf and stem, respectively. Plant nitrogen use efficiency (NUE) was calculated as the ratio of plant biomass to N uptake

### 4.4. Statistical Analyses

The data of Ψ_l_, ΔDM, LA, SLA, WU, WUE, N content, and NUE were assessed using a two-way analysis of variance (ANOVA). The main factor effects of the PGPR (*B.p.*) and irrigation treatment (I), as well as their interaction effect, were analyzed using the SPSS version 18.0 (IBM, Electronics). When *p* < 0.05 by Tukey’s test, the differences between treatments were considered to be significant. Once the interaction was significant, Duncan’s multiple range test was conducted.

## 5. Conclusions

In conclusion, DI had greater effects on leaf gas exchange, plant growth, WUE, and nutrient uptake compared to *Bacillus pumilus* (*B.p.*) inoculation. Specifically, DI restricted leaf gas exchange rates decreased leaf water potential, weakened nutrient uptake, decreased leaf area and plant dry matter (DM), indicating DI inhibited plant growth. The reduction in tomato plant water use under the DI strategy was greater than that in DM, resulting in elevated WUE at the plant scale. Furthermore, *B.p.* inoculation in synergy with DI significantly affected leaf gas exchange rates, SD, and endogenous ABA levels in leaf, and plants inoculated with *B.p.* at 20% soil water content possessed the highest WUE at stomatal and leaf scales, enabling plants to utilize water more efficiently under soil water deficit conditions. These findings provide important insights into the mechanisms of soil water limitation and PGPR applications in tomato plants.

## Figures and Tables

**Table 1:** Photosynthetic rate (A_n_), stomatal conductance (g_s_), transpiration rate (T_r_), intrinsic water use efficiency (WUE_i_), instantaneous water use efficiency (WUE_T_), stomata density (SD), leaf water potential (Ψ_l_), leaf area (LA), specific leaf area (SLA), dry matter of leaf (DM_leaf_) and stem (DM_stem_) and total dry matter (DM) as affected by *Bacillus pumilus* (*B.p.*) inoculation at first harvest, when all plants grew 2 weeks after irrigated to field capacity. Non-*B.p.* control means plants free from *B.p.* inoculation. The data in the table were expressed as mean ± standard error (SE), n = 4. Student’s *t*-test was applied. * indicates significance level at *p* < 0.05; ns denotes no significance.

Treatment	A_n_	g_s_	T_r_	WUE_i_	WUE_T_	SD	Ψ_l_	LA	SLA	DM_leaf_	DM_stem_	DM
	μmol m^−2^ s^−1^	mol m^−2^ s^−1^	mmol m^−2^ s^−1^	μmol mol^−1^	μmol mmol^−1^	mm^−2^	MPa	cm^2^	cm^2^ g^−1^	g	g	g
Non-*B.p.* control	17.97 ± 0.76	0.24 ± 0.02	3.85 ± 0.24	75.35 ± 4.29	4.71 ± 0.24	145.22 ± 13.10	−0.45 ± 0.02	123.16 ± 6.49	326.65 ± 4.51	0.38 ± 0.02	0.13 ± 0.01	0.51 ± 0.03
*B.p.*	17.83 ± 0.40	0.25 ± 0.01	4.07 ± 0.21	71.26 ± 3.93	4.41 ± 0.23	190.94 ± 29.30	−0.42 ± 0.06	119.93 ± 6.09	348.52 ± 7.96	0.35 ± 0.02	0.10 ± 0.01	0.45 ± 0.03
Student’s *t*-test	0.38 ns	0.32 ns	0.27 ns	0.30 ns	0.27 ns	0.13 ns	0.27 ns	0.29 ns	0.08 ns	0.13 ns	0.04 *	0.08

**Table 2:** The effect of *Bacillus pumilus* (*B.p.*) inoculation on leaf ABA ([ABA]_leaf_), stomata density (SD), leaf water potential (Ψ_l)_, leaf area (LA), leaf dry matter (DM_leaf_), specific leaf area (SLA), dry matter (DM), dry matter increment (ΔDM), plant water use (WU) and water use efficiency (WUE) under four irrigation regimes.

*Bacillus pumilus*	Irrigation	[ABA]_leaf_ (ng g^−1^ FW)	SD (mm^−2^)	Ψ_l_ (MPa)	LA (cm^2^)	DM_leaf_ (g)	SLA (cm^2^ g^−1^)	DM_stem_ (g)	DM (g)	ΔDM (g)	WU (L)	WUE (kg m^−3^)
Non-*B.p.* control	I1	74.34 ± 5.52 d	198.22 ± 5.52 b	−0.67 ± 0.02	1814 ± 117	10.58 ± 0.76	171.75 ± 1.60	3.02 ± 0.30	13.60 ± 1.04	13.10 ± 1.04	4.23 ± 0.23	3.09 ± 0.10
Non-*B.p.* control	I2	77.30 ± 3.16 d	193.90 ± 3.16 b	−0.79 ± 0.03	1823 ± 71	10.82 ± 0.35	168.45 ± 1.37	3.37 ± 0.21	14.19 ± 0.54	13.68 ± 0.54	4.32 ± 0.25	3.18 ± 0.07
Non-*B.p.* control	I3	96.19 ± 9.35 bc	186.98 ± 9.35 bc	−0.90 ± 0.09	1494 ± 56	8.95 ± 0.35	167.02 ± 0.39	2.90 ± 0.10	11.85 ± 0.44	11.34 ± 0.44	3.34 ± 0.18	3.40 ± 0.11
Non-*B.p.* control	I4	109.68 ± 3.52 a	176.01 ± 3.52 bc	−0.91 ± 0.05	1394 ± 45	8.51 ± 0.22	163.75 ± 1.90	2.81 ± 0.13	11.31 ± 0.30	10.81 ± 0.30	2.88 ± 0.13	3.76 ± 0.07
*B.p.*	I1	96.53 ± 6.11 bc	166.28 ± 6.11 c	−0.67 ± 0.05	1740 ± 38	10.17 ± 0.14	171.32 ± 5.01	2.77 ± 0.17	12.94 ± 0.30	12.49 ± 0.30	4.01 ± 0.12	3.11 ± 0.04
*B.p.*	I2	99.16 ± 6.15 abc	198.08 ± 6.15 b	−0.70 ± 0.07	1851 ± 86	10.82 ± 0.91	172.72 ± 7.08	3.66 ± 0.27	14.48 ± 1.18	14.03 ± 1.18	4.52 ± 0.33	3.10 ± 0.04
*B.p.*	I3	93.12 ± 12.89 c	222.24 ± 12.89 a	−0.80 ± 0.08	1525 ± 76	8.76 ± 0.87	176.91 ± 9.76	2.98 ± 0.36	11.74 ± 1.23	11.29 ± 1.23	3.49 ± 0.34	3.23 ± 0.06
*B.p.*	I4	105.44 ± 10.17 ab	187.98 ± 10.17 bc	−0.85 ± 0.02	1353 ± 93	8.64 ± 0.84	158.01 ± 6.53	3.11 ± 0.25	11.75 ± 1.08	11.30 ± 1.08	3.01 ± 0.23	3.70 ± 0.07
		*p* value of significant test										
PGPR		0.002 **	0.386	0.134	0.796	0.796	0.597	0.547	0.982	0.973	0.676	0.179
SWC		<0.001 ***	0.019 *	0.004 **	<0.001 ***	0.003 **	0.139	0.05 *	0.01 **	0.01 **	<0.001 ***	<0.001 ***
PGPR × SWC		0.001 **	0.002 **	0.784	0.874	0.974	0.505	0.633	0.917	0.917	0.806	0.592

Note: Non-*B.p.* control and *B.p.* indicated plant’s roots were not or were treated with *Bacillus pumilus* solution, respectively. I1, I2, I3, and I4 indicated plants were exposed to 35% (*v*/*v*) SWC, 30% SWC, 25% SWC, and 20% SWC, respectively. Two-way ANOVA was applied. The data in the table were expressed as mean ± SE, n = 4. Different letters indicate significant difference between treatments according to Duncan’s multiple range test at *p* < 0.05. *, ** and *** indicate significance levels at *p* < 0.05, *p* < 0.01 and *p* < 0.001, respectively.

**Table 3:** The effect of *Bacillus pumilus* (*B.p.*) inoculation on the contents of nitrogen (N), phosphorus (P), and potassium (K) in leaf and stem, and total uptake of N, P, K, and nitrogen use efficiency (NUE) under four irrigation regimes.

*Bacillus pumilus*	Irrigation	[N]_leaf_	[N]_stem_	[P]_leaf_	[P]_stem_	[K]_leaf_	[K]_stem_	Total N	Total P	Total K	NUE
		g kg^−1^	g kg^−1^	g kg^−1^	g kg^−1^	g kg^−1^	g kg^−1^	g plant^−1^	mg plant^−1^	g plant^−1^	g g^−1^
Non-*B.p.* control	I1	34.62 ± 1.04	25.23 ± 0.95	2.45 ± 0.20	2.46 ± 0.18 ab	26.95 ± 0.97	70.75 ± 2.16	0.44 ± 0.04	33.77 ± 4.84	0.50 ± 0.05	30.78 ± 0.66
Non-*B.p.* control	I2	35.85 ± 0.99	24.43 ± 0.43	2.48 ± 0.09	2.32 ± 0.03 ab	29.42 ± 1.32	70.30 ± 2.06	0.47 ± 0.02	34.58 ± 0.72	0.56 ± 0.03	30.21 ± 0.64
Non-*B.p.* control	I3	33.48 ± 1.29	25.52 ± 1.01	2.44 ± 0.10	2.56 ± 0.07 a	28.92 ± 1.02	65.82 ± 2.19	0.37 ± 0.02	29.34 ± 1.70	0.45 ± 0.02	31.79 ± 0.87
Non-*B.p.* control	I4	30.59 ± 0.70	24.26 ± 0.73	1.80 ± 0.07	1.80 ± 0.02 d	27.66 ± 1.16	57.07 ± 1.92	0.33 ± 0.01	20.34 ± 0.51	0.39 ± 0.01	34.50 ± 0.59
*B.p.*	I1	36.22 ± 1.22	29.15 ± 0.38	2.53 ± 0.09	2.59 ± 0.09 a	30.26 ± 0.71	72.18 ± 0.92	0.45 ± 0.02	32.91 ± 1.85	0.51 ± 0.02	28.89 ± 0.72
*B.p.*	I2	35.12 ± 1.12	26.53 ± 0.82	2.61 ± 0.12	2.49 ± 0.13 a	30.67 ± 0.64	66.56 ± 0.92	0.48 ± 0.03	37.17 ± 2.82	0.58 ± 0.05	30.40 ± 0.65
*B.p.*	I3	35.22 ± 1.61	26.34 ± 0.75	2.46 ± 0.19	2.17 ± 0.11 bc	30.71 ± 1.61	69.20 ± 3.51	0.38 ± 0.03	27.44 ± 1.16	0.47 ± 0.03	30.43 ± 1.09
*B.p.*	I4	29.35 ± 1.39	25.54 ± 0.93	1.84 ± 0.15	1.93 ± 0.06 cd	26.14 ± 0.38	55.42 ± 1.30	0.33 ± 0.03	21.98 ± 2.62	0.40 ± 0.03	35.40 ± 1.11
		*p* value of significant test									
*B.p.*		0.691	0.001 ***	0.508	0.849	0.116	0.923	0.754	0.832	0.627	0.361
SWC		<0.001 ***	0.046 *	<0.001 ***	<0.001 ***	<0.023 *	<0.001 ***	<0.001 ***	<0.001 ***	<0.001 ***	<0.001 ***
PGPR × SWC		0.489	0.232	0.978	0.022 *	0.163	0.327	0.999	0.770	0.992	0.306

Note: Non-*B.p.* control and *B.p.* indicated plant’s roots were not or were treated with *Bacillus pumilus* solution, respectively. I1, I2, I3, and I4 indicated plants were exposed to 35% (*v*/*v*) SWC, 30% SWC, 25% SWC, and 20% SWC, respectively. Two-way ANOVA was applied. The data in the table were expressed as mean ± SE, n = 4. Different letters indicate significant difference between treatments according to Duncan’s multiple range test at *p* < 0.05. * and *** indicate significance levels at *p* < 0.05 and *p* < 0.001, respectively.

## Data Availability

Not applicable.
